# Women’s perceptions of factors influencing their food shopping choices and how supermarkets can support them to make healthier choices

**DOI:** 10.1186/s12889-021-11112-0

**Published:** 2021-06-05

**Authors:** Preeti Dhuria, Wendy Lawrence, Sarah Crozier, Cyrus Cooper, Janis Baird, Christina Vogel

**Affiliations:** 1 Medical Research Council Lifecourse Epidemiology Unit, University of Southampton, Southampton General Hospital, Tremona Road, Southampton, SO16 6YD UK; 2 National Institute for Health Research Southampton Biomedical Research Centre, University of Southampton and University Hospital Southampton NHS Foundation Trust, Tremona Road, Southampton, SO16 6YD UK

**Keywords:** Supermarket, Food shopping choices, Women, Qualitative methods

## Abstract

**Objectives:**

To examine women’s perceptions of factors that influence their food shopping choices, particularly in relation to store layout, and their views on ways that supermarkets could support healthier choices.

**Design:**

This qualitative cross-sectional study used semi-structured telephone interviews to ask participants the reasons for their choice of supermarket and factors in-store that prompted their food selections. The actions supermarkets, governments and customers could take to encourage healthier food choices were explored with women. Thematic analysis was conducted to identify key themes.

**Setting:**

Six supermarkets across England.

**Participants:**

Twenty women customers aged 18–45 years.

**Results:**

Participants had a median age of 39.5 years (IQR: 35.1, 42.3), a median weekly grocery spend of £70 (IQR: 50, 88), and 44% had left school aged 16 years. Women reported that achieving value for money, feeling hungry, tired, or stressed, and meeting family members’ food preferences influenced their food shopping choices. The physical environment was important, including product quality and variety, plus ease of accessing the store or products in-store. Many participants described how they made unintended food selections as a result of prominent placement of unhealthy products in supermarkets, even if they adopted more conscious approaches to food shopping (i.e. written or mental lists). Participants described healthy eating as a personal responsibility, but some stated that governments and supermarkets could be more supportive.

**Conclusions:**

This study highlighted that in-store environments can undermine intentions to purchase and consume healthy foods. Creating healthier supermarket environments could reduce the burden of personal responsibility for healthy eating, by making healthier choices easier. Future research could explore the interplay of personal, societal and commercial responsibility for food choices and health status.

**Supplementary Information:**

The online version contains supplementary material available at 10.1186/s12889-021-11112-0.

## Introduction

The frequent intake of energy dense foods, high in fat, sugar, and salt, and infrequent intake of fruit, vegetables and wholegrains are important risk factors for obesity and non-communicable diseases (NCDs), such as type 2 diabetes, cardiovascular disease and cancer [1, 2]. Poor diet, obesity and NCDs are not equally distributed, with the greatest burden falling on those most disadvantaged [3]. Exposure to NCD risk factors can commence early in life, even before conception [4]. Women, particularly those from more disadvantaged backgrounds, are therefore an important target group for intervention and research because they remain predominantly responsible for household food tasks and their food choices impact the short and long-term health of their children [5, 6]. Interventions that show the greatest potential to reduce dietary inequalities are those that address the environmental and social determinants of diet [7].

Glanz and colleagues conceptualised the environmental determinants of diet in their model of nutrition environments. This model is based on an ecological approach to health, and incorporates policy, environmental, psychological influences on diet [8]. The environmental focal point defines four distinct settings that can directly and indirectly influence dietary behaviours, namely: information (media/advertising), organisational (school/work), community (food outlet type and location) and consumer (in-store environments). Each of these settings has developed into their respective field of research and it is now widely recognised that these physical environments can have considerable influence on an individual’s food choice [9, 10].

There is now widespread interest in targeting supermarkets as a setting to improve population diet because of their potential to reach large numbers of customers simultaneously [11]; in the UK, 89% of consumers report buying their everyday food and drink products from major supermarket chains [12]. Within supermarkets, a range of in-store environmental factors can influence customers’ food purchasing and dietary patterns [8]. Systematic reviews of the effects of supermarkets on diet-related behaviours show that current in-store environments tend to encourage purchasing and consumption of energy dense products [13–15]. In particular, there is evidence across high income countries that price promotions are more frequent and shelf space is greater for unhealthy than healthy foods, and that unhealthy products are typically positioned in prominent in-store locations such as checkouts or aisle-ends [16–18]. Collectively this body of evidence suggests that less healthy supermarket environments may be contributing to poor diet because appreciable cognitive effort is needed to make healthier dietary choices [19].

According to the dual process model, food shopping can be described in terms of being governed by two systems: automatic and unintentional, or reasoned and analytical [20]. This model has been somewhat supported by previous research which found that shopping at less healthful discount supermarkets, with poorer availability, pricing and positioning of healthy foods, was more detrimental for the dietary quality of women from disadvantaged than advantaged backgrounds [21, 22]. Additionally, parents of young children report substantial difficulty food shopping when their children are consistently faced with unhealthy food products placed at child-friendly heights and in unavoidable locations, such as checkouts [23, 24]. These studies suggest that current supermarket environments may be particularly influential for those who are more reliant on automatic decision making processes.

Previous qualitative research shows that consumers are supportive of supermarkets doing more to make buying healthy products easier [23, 25, 26]. This prior work, however, provided limited investigation into what these changes could entail, mainly focusing on restricting unhealthy products at checkouts. Nor did they consider the level of support and commitment required from different stakeholders to improve food shopping behaviours. An enhanced understanding of the perceived in-store influences on the food choices of regular discount supermarket shoppers, and strategies they believe would promote healthful food shopping, could aid development of supermarket interventions supportive of healthy eating that are acceptable by customers. Such findings would be of benefit to policy makers, practitioners, researchers, and community organisations [27].

To gain this in-depth understanding a qualitative approach was used to answer the following research questions:

I. *What are women’s perceptions of factors that influence their supermarket food shopping choices?*
II. *How do women feel they could be supported in making healthier food shopping choices?*

## Materials and methods

### Setting and ethics

This study formed a part of an intervention study that assessed the effects of a healthier supermarket layout on women customers purchasing and dietary patterns. Full details of the intervention study methods are available elsewhere (Vogel et al., under review). In brief, the setting was a UK discount supermarket chain and the study recruited women customers, aged 18–45 years, who regularly shopped in three intervention and three matched-control stores located across England. Participants were not informed about the supermarket intervention but were told the study aimed to assess how women customers’ shopping and dietary patterns change over time. Cross-sectional qualitative data were collected from a sub-group of participants who completed all follow-up waves of the intervention project. This paper reports the findings from these qualitative data. The study was approved by the University of Southampton Faculty of Medicine ethics committee (Ethics ID 20986), and abides by the Declaration of Helsinki, Research Governance Framework for Health and Social Care and Data Protection regulations. Reporting of this study follows COnsolidated criteria for REporting Qualitative research (COREQ) recommendations [28].

### Participants

Women were randomly selected, using a random number table, from the 129 participants who had provided complete quantitative data. We aimed to recruit equal numbers of participants from the intervention and control arm of the study and a total of 26 women (*n* = 13 intervention and *n* = 13 control) were approached. They were sent a letter inviting them to participate in a telephone interview and offered an incentive (£10 Love2Shop voucher) upon completion of the interview. Women were telephoned within a week of the letters being posted. If no response was received after six attempts to contact the women, they were considered to have declined. Interested women provided their informed consent to participate in this qualitative sub-study by phone. Participants were informed that they had the right to withdraw from the study at any time and permission to record the telephone interviews was also sought.

### Interviews

Before commencing the study, a literature review was conducted to identify conceptual models that could aid understanding of the mechanisms by which supermarket environments influence dietary choices and to examine existing qualitative evidence of how women perceive supermarket environments affect their purchasing/dietary patterns [23–25, 29]. An initial interview guide was developed to provide responses on gaps in the existing literature. These questions were piloted to enable refinements to be made to the semi-structured discussion guide (supplementary material) and for junior researchers (SF, DPN) to gain experience conducting telephone interviews with training and support from experienced qualitative researchers (WTL, CV). A semi-structured discussion guide was used because it allows the interviewer to explore the topics of interest systematically and comprehensively, while still enabling participants to direct the discussion and raise specific issues relevant to them [30]. Only the participant and researcher were present during the telephone interviews. Participants were not shown the questions in advance and no notes were taken.

### Statistical analysis

Descriptive variables were calculated as percentage (frequency) for categorical variables and median (interquartile range) for non-normally distributed continuous variables. Differences between intervention and control participants were tested using chi-squared tests for categorical variables and Mann-Whitney rank sum tests for non-normally distributed continuous variables.

### Thematic analysis

Audio-recordings were transcribed verbatim, with all personal details removed. QSR NVIVO Software 11 was used to organise and analyse the data. Participants did not comment on their transcripts. Although the model of nutrition environments and the dual process model were used to develop the interview guide, inductive thematic analysis was conducted following established guidelines to ensure that themes and sub-themes were derived from the raw data and to provide the possibility for themes inconsistent with these models to emerge [31]. Two researchers (PD, SF) read and familiarised themselves with the data and identified initial codes. The codes were organised into themes and sub-themes to create an initial framework, which was then refined through coding of each transcript; new themes arose during this process. The coding framework was further refined via double-coding of six transcripts (CV, WL). The research team met to discuss the validity of the themes and to agree a final, comprehensive coding framework that represented the interview findings. This approach is aligned with a relativist ontological and subjective epistemic position which purports that reality is a matter of individual perspective and based on personal experience and insight [32]. Each transcript was re-coded to the final coding framework.

Themes and sub-themes were compiled together with verbatim quotations. The analysis considered differences between intervention and control groups.

## Results

A total of 20 women (intervention arm *n* = 10 and control arm *n* = 10) consented to participate and were interviewed between September 2017–April 2018. Fifteen interviews were conducted by SF (MSc, Nutritionist, female) and a further five were conducted by DPN (MSc, Research Assistant, male). The interviews lasted between 11 and 33 min. As shown in Table 1, the average age of participants was 39.5 years, most women were of white ethnicity (95%) and were living with children (80%). Almost half of the women had no formal educational qualifications beyond age 16 (44%) and most were living in more deprived neighbourhoods (80%). There were no significant differences in demographic characteristics between participants in the intervention and control groups, except that women in the control group were more likely to be in paid employment than intervention group women (Table 1).

**Table 1:** Demographic characteristics of participants by group

Characteristic	Total (***n*** = 20)	Control (***n*** = 10)	Intervention (***n*** = 10)	***P***-value
Age (years), median (IQR)	39.5 (35.1, 42.2)	40.8 (35.7, 43.3)	37.2 (32.9, 40.4)	0.17
White ethnicity, % (n)	95% (19)	90% (9)	100% (10)	0.31
Married, % (n)	50% (10)	40% (4)	60% (6)	0.59
People living in house, median (IQR)	4 (3,4)	4 (2,5)	4 (4,4)	1.00
Living with child aged under 18 years, % (n)	80% (16)	70% (7)	90% (9)	0.26
Low education (no qualifications beyond age 16), % (n)	44% (8)	33% (3)	55% (5)	0.78
Most deprived half of the neighbourhood (IMD), % (n)	80% (16)	80% (8)	80% (8)	0.32
Paid employment, % (n)	70% (14)	90% (9)	50% (5)	0.05
Pounds (£) spent on food per week, median (IQR)	70 (50, 88)	58 (45, 80)	78 (60, 100)	0.27

The themes that emerged from the analysis are described below as they address each of the two research questions and illustrated with quotes.

### Research question 1: What are women’s perceptions of factors that influence their supermarket food shopping choices?

Women’s perceptions of factors that influence their supermarket food shopping choices were captured in four themes: *3.1.1) Physical environment, 3.1.2) Value for money, 3.1.3) Influence of family, 3.1.4) Physiological/psychological state.* An additional theme that appeared to underlie all the other themes was *3.1.5) Level of awareness of food decisions in supermarkets*. Even though inductive thematic analysis was conducted, the first four themes could be seen to correspond to the environmental and psychosocial components of the model of nutrition environments [8], and the fifth theme relates to the dual process model in terms of participants’ level of awareness of their decisions [20]. No differences in these five themes were apparent between the intervention and control groups as illustrated below through quotes from interviewees from both groups.

#### Physical environment

Participants reported several practical and physical factors that influence their choice of, and decisions within, the supermarket. Accessibility was reported as a primary reason for using a particular store and was described as: living in close proximity to the supermarket or it being easily accessible by public transport; the supermarket offering free delivery or ‘click and collect services;’ or the location fitting with their normal routine, such as school runs.

*“just the convenience that they are just up the road.” – P043 Con*

*“probably what’s easiest to get to, because I don’t drive, so somewhere accessible by bus.” – P074 Int*

Many women commented that a logical store arrangement and wide aisles allowed for easy navigation and identification of the products they planned to purchase. Participants did not enjoy having to ‘hunt’ for items and stated that when they could not easily find their desired product, they were more likely to purchase unplanned products. Participants described how product placement strategies influenced their food shopping choices, including the purchase of healthy foods.

*“they have just got all their fruit and veg and fresh stuff right at the front of the store as soon as you walk in, it is on your right hand side as you go in, and supermarket B do the same now, and so do supermarket C. Whereas when you go to supermarket D in the town, all their fresh stuff is right at the back in the middle so you kind of think a lot of people just by-pass it because they don’t have to walk through it" – P066 Int*

Women reported disliking supermarkets’ propensity to place unhealthy products in prominent locations that were tempting for children, which exacerbated an already difficult situation for some mothers in resisting their children’s requests.

*“I’d rather it not be right at the checkout. I think I’d rather get them chocolates if they have been good or something, so I don’t like them seeing it there or while we are waiting, they might get irritated if I said no.” – P043 Con*

*“there’s always sweets at the checkout area when you’ve got your kids obviously it’s ‘oh can I have this, can I have that’ you know, the supermarkets aren’t daft.” – P010 Int*

Product quality, choice and variety, particularly for fresh products, were important for some customers in determining which supermarket they chose to shop at.

*“it could be more variety, yeah, I think if the store was a bit bigger they would ‘cause when I go to other A stores, like I will go to London, their variety of salads is more bigger than the one here.” – P011 Con*

*“plus, they’ve got more of a choice as well, so that was good. It does make it more appealing to go in and buy fresh stuff in there. It’s been updated and more produce has made it a hell of a lot better.” – P066 Int*

#### Value for money

All participants mentioned the important role food prices have on their shopping decisions. For many women, the price came before other considerations such as convenience, proximity, and time. Participants talked about seeking value for money both at a product price level and in relation to the overall cost of a shopping trip or their perception of food prices in different supermarket chains.

*“there’s certain places that sell things cheaper, yeah, so I’ve got a kind of like a routine, do you know that I can get my meat and that, in supermarkets D and E, which is much cheaper than going to supermarkets F or A.” – P011 Con*

*"at supermarket E they do the ehm, oh, I can’t think what it’s called, but it’s the Famous Five or something every week, or Super Five, each week they have a particular 5 fruit and vegs on offer for 50p that week" – P137 Int*

Participants spoke with pride and satisfaction at finding good deals and reported that many of their food choices were prompted by in-store price, multi-buy, and introductory offers. Some participants bought their usual food items in bulk if they were on offer to get better value for their money.

*“but then we sort of, you know, bargain hunt and bulk buy if things are on offer.” – P108 Con*

*“if they’re things that we like, and we use, like, say three for twos or buy one get one free, or there’s money off, then we’ll go towards them. Um, that can be quite an incentive actually, if it’s an introductory offer as well” – P041 Int*

#### Influence of family

A number of participants reported that the health of their family members, particularly their children’s health, motivated their food choices. Healthy choices, however, had to be balanced against ensuring they bought food consistent with their family members’ food preferences for less healthy foods.

*"yeah we try and eat relatively healthily in this house. Ehm, especially since having kids I try and set up a bit more of a good impression of like having our 5 a day of fruit and veg and things but ehm, we are not like obsessive about it. I mean, everything in moderation, I think. But yeah, it is important to us, sort of, eating healthy." – P137 Int*

Most women commented that their children caused them to spend more money and buy more food, particularly unhealthy foods, when they attended food shopping trips. Children’s requests were frequently in response to environmental prompts and caused participants to shift from planned purchases to making impromptu purchases.

*“I prefer to do it [shop] on my own because then I know what I’m doing, and I spend less! Less when they’re not around.” – P025 Con*

*“you know I just stood there waiting, and you can just see like they put sweets at the tills and then while my little boy is sat in the pram or in the trolley he’s like ‘can I have that, and can I have that?’ I think that you do tend to pick up things that you wouldn’t normally buy, but because they are stood in front of you, you think oh well, I will just get one, that’s what it’s there for.” – P066 Int*

#### Physiological/psychological state

Participants reported that their food shopping choices can be driven by their mood or general state of mind at the time. Some participants reported spending more on food if they were hungry, particularly when faced with visual and aromatic environmental prompts, and said they made less healthy food choices that they had not planned to make.

*“so, the smell of croissants, if you walk in there and you’re hungry, yeah, the first thing you’re going to think is ‘ooh I’ll have a bit of that!’” – P011Con*

*“yeah I mean, when there’s sweets there, that’s always, if you’re feeling a bit tired and you think aah I’ll have a bar of chocolate before I get home for my tea.” – P137 Int*

#### Level of awareness of food decisions in supermarkets

A recurring theme that appeared to underlie all the other themes was women’s level of awareness of their own decisions whilst supermarket shopping; for many participants this fluctuated throughout a shopping trip. Most women described their approach to food shopping as being conscious and planned. For example, utilising physical or mental shopping lists based on their budgets, products required for specific recipes or to replenish stock at home. This approach was mostly considered to be cost-effective, time-efficient and led to healthier food purchasing.

*“um, just ask the family what they fancy eating during the week, and then write the shopping list from there, and only buy what I have to buy.” – P025 Con*

*“we write a menu at the beginning of the week, so depends on what’s on that menu, where I would buy the things from.” – P112 Int*

 Making unplanned purchases were frequently reported but most women described being conscious of making these purchases. For example, participants remembering, upon seeing an in-store prompt such as a price promotion, that a ‘usual’ household food item was needed at home.

*“say if I’ve forgot to put something on my list that I realise we’re running short of” – P137 Int*

Most women claimed, initially, to never deviate from approaches to shopping of which they were highly aware, although they often gave conflicting information later in the interview, as evidenced below by two pairs of quotes from two different participants.

*“I normally do it you know, write a shopping list and I literally just stick to that, I am quite good at it”– P218 Con*

*“If there’s something at a checkout then I might grab it if I look at it and think ah yeah, like, sometimes in A they’ve always got like nice cakes there or something, so I always end up buying them.” – P218 Con*

*“I have a weekly food chart that swaps every two weeks…. usually vegetables first, because they are at the front, then the cupboard and then frozen and fridge.” – 074 Int*

*“I guess if there’s something different in an aisle I usually go down, I might buy it.” – 074 Int*

These purchasing behaviours, of which women were less aware about, were usually reported later in the interviews after women had reflected on their shopping habits. Unconscious habitual buying described a regular shopping routine, for example, navigating up and down the store aisles and grabbing items from the shelves without much thought. Participants described being less aware of their decision-making and had to think hard to be able to describe these experiences.

*“I think I go by habit really, what I know. I tend to buy the same things” – P043 Con*

The final approach was characterised by unconscious spontaneous shopping choices that were driven almost entirely by environmental prompts and were unplanned. Women recognised that prominent placement, and promotions, of products, both unhealthy and healthy, within the supermarket encouraged this type of purchasing.

*“the fruit and the vegetable being at the front of the store kind of changes what you buy afterwards because you might plan meals around that.” – P025 Con*

*“P: Well normally I have noticed that the special offers as you walk through the doors, it’s something that you see as you walk through the doors so it’s the first thing that you come in contact with.*

*I: And would that encourage you to buy?*

P: Yes yeah. I feel like a child, I am afraid. *laughs*”– P107 Int

### Research question 2: How do women feel they could be supported in making healthier food shopping choices?

Women’s perceptions of strategies that would support them and other people to make healthier food choices while food shopping in supermarkets were categorised under one theme: *3.2.1) Responsibilities: government, supermarkets and personal.* This theme arose from inductive thematic analysis and does not easily conform to either of the conceptual models underpinning this study. No differences in this theme were apparent between participants in the intervention and control groups.

#### Responsibilities: government, supermarkets and personal

While participants described healthy eating as primarily their personal responsibly, some mentioned strategies that governments and supermarkets could support customers to make healthier food choices. The role of nutrition education and labelling were identified as two important areas that were viewed as the responsibility of government.

*“teach people about healthier living. Because I do not think a lot of people know what healthy living actually means and how to eat well” – P001 Int*

*“I mean the packaging is also a little bit overwhelming sometimes, and everybody’s got different versions of how they’re telling you that’s really horrific and loads of fat and salt, because traffic light systems mean different things in different stores, and that’s confusing.” – P041 Int*

When asked about what entailed a healthy diet, however, most participants demonstrated an understanding that it is important to eat fruit and vegetables frequently and processed foods infrequently.

*“having a balanced diet with fruit and veg in it, and not eating too many unhealthy takeaways and fatty food and fizzy drinks.” – P043 Con*

Very few women indicated the need for stronger government intervention on labelling, advertising of unhealthy foods and easy access to takeaway foods.

*“there seems to be take-outs everywhere, there are just too many, I think. Far too many. The fruit and veg stalls or markets all seem to be shutting down” – P010 Int*

The few women that suggested strategies supermarkets could take, mentioned improving the price, promotion and placement balance between healthy and unhealthy products or offering a better range of healthy meal options which were quick and convenient to cook.

“ *rather than having so much of the you know, kind of, the unhealthy stuff on offer, all of the healthy foods on promotion and that kind of thing.” – P108 Con*

*“they could put the fruit at the counters and make it more accessible and attractive. They don’t, it’s all sweets and things like that, crisps and stuff, you don’t see apples and bananas and oranges that easy accessed.” – P 041 Int*

*“I think having a good selection of healthy quick foods you know is helpful.” – P108 Con*

A number of women acknowledged that while supermarkets could better support customers to make more healthy choices, they are ultimately businesses, driven by commercial interests.

*“how they do their advertising and what they put … as you walk in, and the smell of the food... at the end of the day they want customers to spend.” – P011 Con*

*“the supermarket’s aren’t silly, you know, they put things at the front where they know children will spot it straight away … they’re just trying to make money aren’t they?” – P010 Int*

Women consistently described feeling that it was their personal responsibility to make healthy food choices for themselves and their families and described healthy eating in terms of buying and cooking fresh foods. A number of participants expressed the view that they needed more self-control, and to be better organised and plan ahead to ensure they made healthy food shopping, cooking and eating choices.

*“if I choose to go in there and buy a packet of sweets, yeah, that is my choice, and I have to realise yeah the consequences of me eating that packet of sweets, right, that I’m going to maybe gain a few pounds.” – P011 Con*

*“I think at the tills. You know I just stood there waiting, and you can just see like they put sweets at the tills…..Mind you, it’s self-control as well, I suppose!” – P066 Int*

*“…need to be more organised. It’s not just, you know the kind of secret is to plan and know what you need for the week, then it will be more economical, more practical and better for you. But that doesn’t always work in reality!” – P041 Int*

Although women felt a desire and responsibility to cook healthy meals from scratch, they indicated that time, work and family pressures often prevented them from doing so.

*“like Change4Life the kids have brought stuff home from school and they have got healthy recipes in it is just having the time to look at the recipe, to go out and buy the ingredients and then go home to make it. If you get what I mean, it is a process isn’t it, to be able to get there.” – P 172 Con*

*“but, we both work full time, little one goes to school, and the reality is you can’t always guarantee that we’ll all sit down and enjoy a healthy home cooked meal.” – P041 Int*

Some participants also commented that even though they have good intentions of eating healthily, it can be difficult to keep their resolve to eat healthily when faced with temptations in the supermarkets. Conversely, healthy environmental prompts can support eating healthy ambitions.

*“oh actually you see something that catches your eye and you think ‘oh actually, I’ll have that’ or you smell the cakes in the bread department because there are some beautiful smells and you think ‘oh one cake is not going to hurt’ and then you buy it and when you get home you think, oh I really shouldn’t have brought that but oh well never mind I’ve got it, I may as well eat it and it is just too late.” – P 139 Con*

*I’ve pulled into D petrol station, they had bananas on the counter, and I grabbed one because I thought actually, I don’t want a bar of chocolate but I needed something. But it would have been, probably before I would have grabbed that [chocolate bar]. –P041 Int*

## Discussion

### Principal findings

The findings of this qualitative study identified that women aged 18–45 years openly conveyed that their shopping habits were driven primarily by an internal desire to select foods that would enable them to make sensible choices for themselves and their families. They acknowledged that the supermarket environment influenced their food shopping choices, but felt this was primarily due to them making conscious choices in terms of value for money, product range and quality, and supermarket accessibility. The role of the supermarket environment in generating less conscious food purchasing choices was evident. Participants frequently expressed a conscious desire to make healthy shopping choices and planned to do so by making lists and/or meal schedules. They also reported, however, that the supermarket environment presented cues which led them to make unhealthy decisions that they were less aware of. Prominent placement of products within supermarkets in particular, arose as a key driver of unintended unhealthy food purchasing. Shopping with children posed a challenge for most women who admitted the placement of unhealthy foods within the sight and reach of their children exacerbated their reactions and ultimately influenced what the women bought. Promotions on products aligned to family food preferences also triggered unplanned or bulk purchases. Physiological and psychological state, particularly stress, fatigue, or hunger, while shopping appeared to heighten responses to environmental cues, such as the smell or sight of unhealthy foods, and subsequently altered planned purchases.

Women’s suggestions for strategies to support healthier food shopping patterns reflected their personal values and priorities. Those that prioritised value for money emphasised that reducing the cost of, and increasing the promotions on, healthy foods would prompt them to buy more. Others who felt that meal planning, willpower and individual control were important for healthy shopping practices believed that better nutrition education and labelling would enable people to improve their choices. Across all participants there was a sense of personal responsibility to make healthier food shopping choices for themselves and their families despite the environmental barriers they encountered in the store. While there were suggestions for how supermarkets and governments could support healthier shopping choices, the commercial interests and economic benefits to the supermarkets were presented as a justification for not intervening.

### Comparison with previous literature

Seeking value for money has consistently been described as a dominant factor driving food purchasing decisions, particularly among individuals of lower socioeconomic status [33]. It also arose as a strong influencer of food selection for women in this study, who predominantly lived in more deprived neighbourhoods and had lower educational attainment. While not having adequate or affordable access to healthy foods has been raised as a concern by low income groups in the US, [34] this was not an issue among the group of British women interviewed in this study who shopped in discount supermarkets. This finding may relate to quantitative evidence showing no difference in food accessibility or cost across neighbourhoods of varying deprivation levels in the UK [9, 35].

Participants did not refer to the price of individual products alone but described cost as a complex picture of value for money in relation to other priorities, including family preferences, product variety, convenience, and reward. This concept of cost has been previously identified in both the UK and US [34, 36, 37], and suggests that while fiscal policies, such as taxes on sugary drinks, are important for preventing obesity, their effectiveness would be enhanced if combined with other non-pricing interventions to improve population diet. Participants described achieving value for money by using strategies such as comparing product prices between stores, searching for sale items and buying favoured items in bulk when on promotion. These responses indicate that for customers on more restricted budgets, the physical supermarket environment provides important cues for food selection. Quantitative evidence showing that shopping at large chain or premium supermarkets, which have better availability, pricing and placement of healthy foods than discount supermarkets, can improve dietary quality among women from disadvantaged backgrounds but are less important for the diets of more affluent women supports this interpretation [21, 22].

It has been postulated that a failure to make healthy food decisions occurs when the reasoned system is overthrown by the intuitive system, leading people to fall prey to temptation or act less consciously around food choice [38]. Our analyses of the descriptions of women’s food shopping experiences in this study identified that they did not fit neatly into a dual typology but that competing interactions between conscious processes and unconscious processes activate shoppers’ behaviours. Participants’ food choices in supermarkets were the result of various combinations of the two systems, such as: i) consciously making unintended food purchases, where environmental prompts trigger the need to restock an item or ii) conscious controlled food choices (i.e. picking up items on a written list) being coupled with unconsciously making unintended food purchases (unplanned purchases driven by environmental promotional cues). These findings are consistent with criticisms that the dual process model is an overly simplistic representation of human cognition [39].

Additionally, our findings indicate that these decision making processes can be heightened by other contributing factors, such as psychological or physical state while shopping, or the food preferences of other household members. These findings indicate that the existing model of community nutrition environments could be enhanced to specifically include social factors and physical state. Hunger and stress are known to increase the likelihood of choosing and consuming unhealthy foods. More recent research indicates, however, that food selection is not uniform under these internally-driven conditions and can vary depending on the environmental stimuli; more healthful food choices are made when the food environment directs individuals towards healthy products [40, 41]. Modelling of the pathways outlined in the community nutrition environment model has also shown that less healthy supermarket environments are associated with poorer psychological states and, in turn, with more harmful dietary behaviours [42]. This pathway may be particularly relevant to participants of this study who frequently shopped at discount supermarkets.

Social factors, such as the food preferences of other household members, also provoked a change in participants’ intended shopping patterns. The overwhelmingly unhealthy environmental characteristics of supermarkets increasing the burden of shopping with children has been raised in previous qualitative research [23, 24, 43], however, little effort has been made to quantify the effect different family members have on household food shopping decisions. The results of this qualitative study are being used to design pathway analyses using longitudinal quantitative data to identify the key pathways of influence between environmental, social and psychological determinants of dietary-related factors, and how improving discount supermarket environments effects these pathways [44].

### Policy and research implications

Findings from this study confirm those of others who highlighted that consumers are concerned and often annoyed about the prominent placement and frequent promotion of unhealthy snacks, thus suggesting consumer support for action to limit unhealthy in-store temptations [23]. Overall, however, participants in the current study expressed a level of ambivalence towards the role of supermarkets and governments in altering supermarket environments for health reasons. Women consistently conveyed that the responsibility for making healthy food choices lies with the individual or parent, and that supermarkets had a form of economic entitlement, as any business, to make a profit selling products customers’ desire. This tendency for participants to indicate that individuals have the responsibility for following a healthy diet similarly arose in qualitative discussions conducted with Danish supermarket customers [29]. One possible explanation for this point of view is that individualism is a core part of the culture in many high income countries, making individual responsibility for health behaviours both a deeply-held value and a moral virtue [45].

The cultural tendency towards individualism may be attributable, at least in part, to government policies and interventions over the past two decades that aimed to address obesity and inequalities by focusing heavily on individual behaviour change as the solution [46]. Media coverage has additionally framed obesity as an issue of self-control and self-responsibility, omitting, until quite recently, the way in which private companies and public organisations shape the capabilities of individuals in making food choices [47]. While it is increasingly recognised among health professionals that dietary patterns result from a range of factors, and they acknowledge the commercial determinants of health as an area for policy action [48], the sustained focus on individual behaviour change in government policy essentially neutralises the perceived role of governments and the food industry [46]. It is therefore unsurprising that members of the public feel it is up to them to avoid unhealthy food choices, and are more supportive of information-based interventions by government, such as labelling or dietary guidance, over structural interventions, such as regulations to tax or limit promotions of unhealthy foods [49]. Despite structural interventions showing greater effectiveness at improving dietary behaviours than educational strategies [50], public acceptance of such interventions is currently low, but it is critical for politicians to implement them into policy [51].

Future research, using more novel qualitative methods, such as go-along interviews or narrative interviews with the customers [52, 53], could explore in further depth why many consumers continue to believe that healthy eating is a personal responsibility, rather than a duty of governments and food businesses. Raising consumer awareness of the commercial determinants of obesity and poor diet may be one strategy for future research to mobilise public demand for structural changes to improve supermarket environments. Recent work suggests that communicating the proven effectiveness of an intervention at improving a health behaviour, can increase public acceptance of that intervention [49]. Assessing public acceptance of the UK Government’s intended ban on promoting unhealthy foods in retail outlets is needed to identify if changes in attitudes towards personal responsibility for healthy eating occur following implementation [54].

### Strengths and limitations

This study provides insight into the lived experiences of food shopping for women who frequently rely on discount supermarkets. A methodological consideration is that this study was conducted with participants of an intervention study, however, no differences were seen in the intervention and control groups’ responses, likely because participants were not informed of the intervention. The number of women interviewed in this study was not exhaustive and some interviews were of shorter duration than may have been anticipated, yet the data reached saturation point on the research questions of the study. A different interview method, such as go-along interviews, may have enabled more in-depth information to be received from participants but the geographical spread of participants prohibited use of this method in this study. The views expressed by study participants cannot be generalised to all young women who frequently shop in discount supermarkets in England. Those interviewed nevertheless represent a valuable sample of individuals from more disadvantaged backgrounds and strengthen the qualitative literature base in the field of store placement interventions. Presentation of the qualitative data in this paper is only one of a number of possible interpretations. To ensure the interpretation was a fair representation of participants’ experiences, a rigorous double-coding process was adopted. Any disagreements in coding were resolved through discussion with the full coding team. Statistical analyses were conducted to assess differences in the demographic characteristics of participants from the two study groups. These analyses are important to identify whether these factors could be considered drivers of any differences in responses between the intervention and control groups. The small sample size of the groups hinders the statistical relevance somewhat, yet a statistically significant difference in one characteristic was observed.

## Conclusions

This study identified a number of factors that influence women’s food shopping patterns and described how current placement strategies in supermarkets influence women’s food shopping intentions and choices. While women largely described adopting a disciplined approach to food shopping, the nature of the many unhealthy environmental cues triggered them into making poorer food choices that were unintended. These responses to the supermarket environment were heightened by certain physiological or psychological states, such as hunger or stress, and family food preferences. Supermarkets have an important role in making their environments more healthful to enable customers to consistently make healthy choices. Further exploration of strategies to mobilise public support for healthier supermarket environments is needed to prompt and support policy action.

## Supplementary Information

**Additional file 1.** Semi-structured interview guide for women.

## Data Availability

The coding tree and datasets generated and analysed during the current study are available from the corresponding author on reasonable request.
