# Supplementary material for: Women’s perceptions of factors influencing their food shopping choices and how supermarkets can support them to make healthier choices
Source: BMC Public Health. 2021 Jun 5;21:1070. doi: 10.1186/s12889-021-11112-0 (PMC8178895; doi:10.1186/s12889-021-11112-0)
Supplement: Supplementary file 1 — Additional file 1. Semi-structured interview guide for women. [file 12889_2021_11112_MOESM1_ESM.docx]

**Women’s perceptions of factors influencing their food shopping choices and how supermarkets can support them to make healthier choices**

Preeti Dhuria,^1^ Wendy Lawrence,^1,2^ Sarah Crozier,^1^ Cyrus Cooper,^1,2^ Janis Baird,^1,2^ Christina Vogel^1,2^

^1^ Medical Research Council Lifecourse Epidemiology Unit, University of Southampton, Southampton General Hospital, Tremona Road, Southampton, SO16 6YD, UK

^2^ National Institute for Health Research Southampton Biomedical Research Centre, University of Southampton and University Hospital Southampton NHS Foundation Trust, Tremona Road, Southampton SO16 6YD UK

**Correspondence to:** Preeti Dhuria, pd@mrc.soton.ac.uk, Tel: 023 8076 4042, University of Southampton, Southampton General Hospital, Tremona Road, Southampton SO16 6YD, UK

**Short title:** Factors influencing women’s supermarket food choices

**Keywords:** Supermarket, food shopping choices, women, qualitative methods

**Supplementary material – Semi-structured discussion guides for women**
